# Case Report: Therapeutic effect of efgartigimod in refractory anti-GQ1b antibody syndrome coexisting with myasthenia gravis

**DOI:** 10.3389/fimmu.2025.1605985

**Published:** 2025-06-10

**Authors:** Keiko Watanabe, Seiya Takahashi, Akane Kanda, Takuya Watanabe, Yuki Kakinuma, Satoshi Yano, Ryuta Kinno

**Affiliations:** ^1^ Department of Internal Medicine, Showa Medical University Northern Yokohama Hospital, Yokohama, Japan; ^2^ Department of Neurology, Showa Medical University Fujigaoka Hospital, Yokohama, Japan

**Keywords:** anti-GQ1b antibody syndrome, autoimmune neuromuscular disorder, myasthenia gravis, efgartigimod, intravenous immunoglobulins, plasmapheresis

## Abstract

Anti-GQ1b antibody syndrome is a spectrum of autoimmune neurological disorders that includes Miller Fisher syndrome, Guillain-Barré syndrome (GBS) with ophthalmoplegia, Bickerstaff brainstem encephalitis, and acute ophthalmoplegia without ataxia. These conditions are characterized by the presence of immunoglobulin G (IgG) antibodies targeting GQ1b gangliosides. The coexistence of anti-GQ1b antibody syndrome and myasthenia gravis (MG) is rare and presents diagnostic and therapeutic challenges. We report the case of an 84-year-old Japanese man with overlapping features of both disorders, describing his clinical course and response to add-on treatment with the neonatal Fc receptor antagonist efgartigimod. He presented with fever and diarrhea, followed by acute limb weakness. He was initially suspected of having had a stroke but was later diagnosed with GBS based on areflexia, anti-ganglioside antibody positivity, and nerve conduction abnormalities. Intravenous immunoglobulin therapy was initiated but his condition worsened, leading to respiratory failure and mechanical ventilation. Subsequently, bilateral ptosis and eye movement dysfunction emerged, prompting the consideration of MG. Anti-acetylcholine receptor antibodies and tensilon test results were positive and high-dose methylprednisolone was administered, resulting in partial improvement. Plasmapheresis was performed, but profound limb weakness and respiratory failure persisted; intravenous efgartigimod was thus introduced. Remarkably, the patient’s respiratory function improved within 7 days, leading to ventilator weaning, and his limb weakness showed notable recovery. After a second cycle of efgartigimod, the patient regained speech and independent mobility, allowing transfer to a rehabilitation facility. His case underscores the diagnostic complexity of overlapping anti-GQ1b antibody syndrome and MG, and it highlights the therapeutic potential of efgartigimod in treating refractory cases of overlapping autoimmune neuromuscular syndromes. Given the rapid efficacy of efgartigimod for improving both respiratory and motor functions in this case, it is apparent that efgartigimod can be a valuable therapeutic option in complex neuromuscular autoimmune conditions.

## Introduction

Anti-GQ1b antibody syndrome is a spectrum of autoimmune neurological disorders characterized by the presence of immunoglobulin G (IgG) antibodies targeting GQ1b gangliosides, which are abundant in the human nervous system. This syndrome encompasses multiple overlapping conditions including Miller Fisher syndrome (MFS), Guillain-Barré syndrome (GBS) with ophthalmoplegia, Bickerstaff brainstem encephalitis (BBE), and acute ophthalmoplegia without ataxia (AO) (1). The coexistence of anti-GQ1b antibody syndrome and myasthenia gravis (MG) is rare but documented, with case reports demonstrating overlapping autoimmune mechanisms and clinical challenges in diagnosis (2–4). Standard treatments such as intravenous immunoglobulin (IVIG) are often effective for both anti-GQ1b antibody syndrome and MG, with reported efficacy in improving ophthalmoplegia and bulbar symptoms (2).

We present the case of an 84-year-old Japanese man who initially exhibited fever and diarrhea, subsequently developing progressive limb weakness and respiratory failure. Despite receiving treatment with IVIG and plasmapheresis, his symptoms persisted. The administration of intravenous efgartigimod, a neonatal Fc receptor (FcRn) antagonist, resulted in significant improvements in both respiratory function and limb strength, indicating its potential efficacy not only for MG but also for anti-GQ1b antibody syndrome characterized by the GBS with ophthalmoplegia. This case underscores the role of efgartigimod as a novel therapeutic option for autoimmune neuromuscular disorders, particularly in refractory cases. Further research is necessary to investigate its broader applications in overlapping syndromes.

## Case presentation

**Figure 1:**
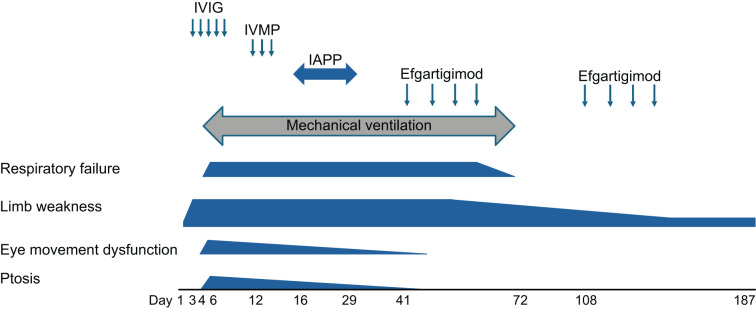
The patient’s clinical course after admission. The timing of each therapy and clinical symptoms are shown. Day 1: the day of admission to our neurology department. Note the significant improvement after the intravenous efgartigimod treatment. IVIG, intravenous immunoglobulin; IVMP, intravenous methylprednisolone.

An 84-year-old Japanese man presented to a local clinic with fever and diarrhea. Four days later, he developed weakness in his right upper extremity, prompting him to seek consultation at our neurosurgical department (day 1; **Figure 1**). The patient was admitted to the hospital on the same day due to the possibility of a cerebral infarction. The following day, weakness also manifested in his left upper extremity and both lower extremities, leading to his transfer to our neurology department (day 3). Upon admission, his percutaneous oxygen saturation was recorded at 97% (room air), and all other vital signs were normal. Manual muscle testing (MMT) revealed a score of 0 for both upper and lower extremities, with no tendon reflexes detected. The patient exhibited reduced touch sensation in the extremities, while vibratory sensation remained intact.

A cranial-nerve examination showed no abnormalities. Initial laboratory findings indicated elevated levels of creatinine (1.21 mg/dL) and C-reactive protein (2.90 mg/dL). The patient was also positive for anti-acetylcholine receptor (anti-AChR) antibodies (0.6 nmol/L) and negative for anti-MuSK antibodies (<0.02 nmol/L). An enzyme-linked immunosorbent assay showed that the patient’s serum IgG reacted with GM1, GD1a, GQ1b, and GalNAc–GD1a complexes with phosphatidic acid (**Table 1**). A cerebrospinal fluid (CSF) examination showed normal findings (cell count 0/μL, protein 27 mg/dL, glucose 81 mg/dL, chloride 125 mEq/L, oligoclonal bands: negative, HSV-PCR <2×10² copies/mL, VZV-PCR <2×10² copies/mL, IgG index 0.49). No evoked potentials were recorded for the bilateral median, ulnar, and tibial nerves during motor nerve conduction studies, whereas normal patterns were recorded during sensory nerve conduction studies (**Table 2**), indicating the presence of the axonal forms of GBS (5).

**Table 1:** Changes in the patient’s antibody from before to after efgartigimod therapy.

Antibody	Before treatment	After IVIG	After IAPP	After efgartigimod
GM1	+++	+++	++	−
GM2	+	−	−	−
GM3	−	−	−	−
GD1a	+++	+++	++	−
GD3	−	−	−	−
GT1b	−	−	−	−
GQ1b	+	++	−	−
Gal–C	−	−	−	−
GalNAc–GD1a	++	+++	−	−
GD1a/GD1b	−	−	−	−
AchR (nmol/L)	0.6	0	0	0
Serum IgG (mg/dL)	1449	N/A	818	597

IgG antibodies against mixed antigens of glycolipids and phosphatidic acid were measured by enzyme-linked immunosorbent assay. According to optical density, the titer was determined semi-quantitatively from - (negative), + (mildly positive), ++ (moderately positive) to +++ (strongly positive). IAPP, immunoadsorption plasmapheresis; IVIG, intravenous immunoglobulin; N/A, not available.

**Table 2:** Sensory nerve conduction study.

Nerve	Latency (ms)	Amp (μV)	Velocity (m/s)
Rt. median	3.2	15.7	49.7
Lt. median	3.4	33.4	45.9
Rt. ulnar	2.6	23.3	46.5
Lt. ulnar	2.7	29.3	51.5
Rt. sural	2.3	6.3	59.6
Lt. sural	2.6	10.5	61.9

Note that no evoked potentials were recorded for the bilateral median, ulnar, and tibial nerves during motor nerve conduction studies. Lt, left; Rt, right.

Based on the clinical symptoms observed, we initially considered a diagnosis of GBS and subsequently administered IVIG therapy (0.4 g/kg/day for 5 days) starting on day 3. However, the patient’s clinical symptoms worsened, leading to the development of dysphasia and respiratory failure. By day 4, the respiratory failure had intensified (PaO_2_: 58.8 mmHg, PaCO_2_: 55.5 mmHg), necessitating mechanical ventilation. On day 6, bilateral ptosis, eye movement dysfunction, and neck weakness became apparent. The eye movement dysfunction and ptosis improved temporarily after an injection of edrophonium was administered; the patient’s tensilon test result was positive.

On day 12, we considered the possibility of coexisting MG and administered intravenous methylprednisolone (IVMP: 1 g/day for 3 days). This treatment resulted in gradual improvement of the patient’s bilateral ptosis and eye movement dysfunction. On day 16, we initiated immunoadsorption plasmapheresis (IAPP) therapy. After seven IAPP sessions, the patient’s neck weakness showed mild improvement, but the limb muscle weakness and respiratory failure persisted. To address the remaining respiratory failure, we commenced the first cycle of intravenous efgartigimod (10 mg/kg/week, with four infusions per cycle) on day 41. Notably, the patient’s lower limb muscle weakness, likely due to GBS, gradually improved, reaching MMT 3 in the lower extremities by day 54 and MMT 1 in the upper extremities by day 68. His respiratory failure also gradually improved, allowing for the resolution of mechanical ventilation on day 72.

After the second cycle of intravenous efgartigimod, the patient became able to speak with a speech valve on day 108 and was transferred to a rehabilitation hospital for further improvement in daily activities on day 187. The patient’s anti-ganglioside and anti-AchR antibodies became negative after intravenous efgartigimod treatment (**Table 1**). Based on the patient’s clinical course, we ultimately diagnosed a case of coexisting anti-GQ1b antibody syndrome and MG that responded positively to add-on therapy with intravenous efgartigimod.

## Discussion

Our patient’s initial symptom was limb weakness followed by ptosis and severe respiratory failure (**Figure 1**). Not only anti-gangliosides but also anti-AChR antibodies were positive. These clinical features and the patient’s positive status for anti-GQ1b-IgG antibodies are typical manifestations of BBE (1, 6). Anti-GQ1b antibodies are the core biomarkers for MFS and BBE and often coexist with other anti-ganglioside antibodies (such as anti-GT1a antibodies), leading to a broader range of neurological symptoms (7–9). Moreover, our patient’s positive status for anti-GM1, anti-GD1a, and GalNAc–GD1a antibodies (**Table 1**) and nonreactive motor nerve-conduction study results with preserved sensory nerve action potentials (**Table 2**) indicated the diagnosis of the axonal form of GBS, presenting as rapidly progressive limb weakness and areflexia (10–12). Fever and diarrhea (likely from a gastrointestinal infection) are common precipitants of GBS/MFS (10, 13). Regarding our patient’s CSF findings, typical GBS may present with protein-cell dissociation, but some variants (such as MFS) may have normal CSF protein (14, 15). Taking these findings together, we consider our case had anti-GQ1b antibody syndrome with multiple overlapping conditions including MFS, axonal form of GBS with ophthalmoplegia, and BBE.

It was difficult to confidently diagnose MG in his case. The clinical symptoms (ptosis, eye movement dysfunction), positive anti-AChR antibodies, and positive on the tensilon test suggest the diagnosis of MG, based on the Japanese clinical guidelines (16). Our patient showed a relatively low titer of anti-AChR antibodies (0.6 nmol/L), which may represent a nonspecific reaction (17). However, his positive tensilon test result indicated neuromuscular transmission dysfunction (18). Other inflammatory neuropathies such as chronic inflammatory demyelinating polyneuropathy (CIDP) and multifocal motor neuropathy were unlikely diagnoses in our patient’s case, based on his clinical course (19, 20). Taking the patient’s findings together, we ultimately diagnosed a case of coexisting MG and anti-GQ1b antibody syndrome with multiple overlapping conditions including MFS, GBS with ophthalmoplegia, and BBE.

After the first-line treatments for MG exacerbation, including IVPM, IAPP, and IVIG (21), our patient’s ptosis resolved but other symptoms persisted. A response to treatment is typically observed within 2 days of plasmapheresis and within 4–5 days of IVIG (22). It has been noted that the therapeutic effect of IVIG may appear over a relatively long period of time, i.e., as long as 28 days (23). Our patient’s limb weakness and respiratory failure persisted even after 40 days of IVIG, which suggested that standard first-line therapies alone were inadequate for his treatment. GBS with anti-GM1 antibodies may have a poorer response to IVIG treatment, especially when axonal damage is significant (11, 24). Furthermore, anti-GQ1b antibody-associated brainstem encephalitis may require higher doses of immunomodulatory treatment (6, 15). Our patient’s worsening condition after IVIG treatment may be related to this. His limb weakness and respiratory failure resolved after intravenous efgartigimod therapy. Although it remains unclear whether our patient’s respiratory failure was due to MG or GBS, the improvement of his limb weakness suggests that add-on therapy with intravenous efgartigimod may be useful not only for MG but also for GBS.

The respective reported incidence of GBS is 0.4–1.7 per million people per year, and that of MG is 10–20 per million people per year (25, 26). The statistical probability of GBS and MG occurring together is <1 in 10 billion, making such cases very rare, and the reported incidence of GBS concomitant with MG is 0.4–1.7 per million people per year (27). One of the reasons for the paucity of reported cases is that GBS and MG present with similar symptoms and neurological findings, making it difficult to diagnose both diseases in a single patient with severe symptoms. Regarding the biological mechanism, we speculate that the coexistence of GBS and MG is theorized to be caused by molecular mimicry of infectious agents and self-antigens. Experimental studies have revealed that IgG antibodies from GBS patients cross-react with AchR in mice, thus demonstrating that a pathogenic activation of the immune system, such as by infection, can lead to the production of cross-reactive antibodies against both myelin protein in peripheral nerves and AchR at the neuromuscular junction (28). It has also been suggested that infection may not only induce autoantibodies that cause GBS; it may also promote the production of AchR antibodies, leading to the development of MG (29).

The treatment of GBS by the administration of plasmapheresis therapy and IVIG has been demonstrated to be effective. The mechanisms of action of these treatments involve the removal of autoantibodies and complement through plasmapheresis and the inhibition of complement activation following the binding of autoantibodies to peripheral nerve antigens by IVIG. Most patients exhibit improvement following treatment with plasmapheresis and IVIG; however, some cases have demonstrated resistance to these therapeutic interventions. Indeed, in a global multicenter prospective cohort study, 68% of the patients with GBS who were unable to walk unassisted had improved GBS disability scores 4 weeks after an initial treatment with either immunotherapy (30).

Efgartigimod, a drug approved for the treatment of the chronic phase of MG, has been shown to be effective when there is no improvement after repeated first-line therapies. This is due to the ability of efgartigimod to competitively inhibit the binding of IgG autoantibodies to FcRn, thereby promoting the autoantibodies’ degradation within lysosomes and decreasing their blood concentration. The observed improvement in symptoms has been reported to occur within a time frame of 1–2 weeks after the injection, and this rapid effect of efgartigimod suggests the potential for efficacy in the treatment of MG crisis (31). Repeated administration of efgartigimod has been reported to reduce total IgG levels by approximately 75% from baseline across all IgG1–4 subclasses (32). Anti-ganglioside antibodies are classified as IgG1 and IgG3 subclasses, exhibiting structural and functional similarities to anti-AChR antibodies. Consequently, a reduction in antibody levels is anticipated. A review of the literature reveals six documented cases in which efgartigimod has been utilized for the treatment of GBS (33–35) (**Table 3**). The disease types exhibited variability, with two out of the six cases diagnosed as acute motor axonal neuropathy. In five out of the six cases, a decrease in IgG levels was observed. The presence of anti-ganglioside antibodies was confirmed in three out of six cases. Following the administration of efgartigimod, a decline in all antibodies was observed. Taken these findings together, it is hypothesized that the diminution in anti-ganglioside antibody levels plays a pivotal role in the clinical amelioration of GBS. In our patient’s case, the add-on treatment with intravenous efgartigimod improved not only his respiratory failure but also his limb weakness. Given the classification of both ganglioside and AchR antibodies as IgG autoantibodies, we speculate that treatment with efgartigimod may be effective not only for MG but also for other IgG-mediated autoimmune diseases. Indeed, recent investigations showed that efgartigimod treatment also exhibited good efficacy in patients with GBS (33, 34). Moreover, a 2023 study described excellent results regarding MG complicated by stiff person syndrome (an anti-GAD antibody-related disease) successfully treated with efgartigimod (36). The specific mechanism of action requires further research.

**Table 3:** Reported cases of GBS treated with efgartigimod.

No	Age	Sex	Symptoms	Prodromal history	Diagnosis	Timing of Efgartigimod	Gangliosides antibody			Serum IgG		Ref.
							Antibody	Before	After	Before	After	
1	56	M	Weakness, dyspnea	Diarrhea	AMAN	Day13	GT1a GD3	++ +++	− −	20.2g/L	6.71g/L	(33)
2	54	M	Eye movement dysfunction, ataxia, weakness, autonomic symptoms, peripheral sensory function impairment	Cold	MFS-GBS	Day7	GT1a GQ1b GD3 GT1b	+++ +++ + +	++ ++ − −	16.2g/L	9.62g/L	(33)
3	27	M	Eye movement dysfunction, ataxia, weakness	None	MFS-GBS	Day25	GQ1b	+	−	51.9g/L	10.7g/L	(33)
4	81	F	Weakness, peripheral sensory function impairment	Diarrhea	GBS	Day1	Negative			N/A	Decreased by 52%	(34)
5	65	F	Weakness, dyspnea, peripheral sensory function impairment	Sore throat and stuffy nose	GBS	Day2	Not collected			N/A	Decreased by 30%	(34)
6	58	M	Weakness, slurred speech, difficulty swallowing	Febrile illness	AMAN	About one month after onset	Negative			N/A	N/A	(35)

Timing of efgartigimod treatments and changes in the patient’s antibody and serum IgG concentrations from before to after efgartigimod therapy are shown. AMAN, acute motor axonal neuropathy; F: female, M: male, MFS-GBS, MFS-GBS overlap syndrome; N/A: not available.

We observed significant decreases of both anti-AchR and ganglioside antibodies in the present patient after the administration of intravenous efgartigimod (**Table 1**), which suggests that efgartigimod may be more effective than plasmapheresis in removing IgG antibodies for patients with GBS+MG and not only MG. It is known that IgG antibodies sometimes increase by overshooting after plasmapheresis in MG crisis patients (37), which may worsen symptoms. Our patient’s case demonstrates that add-on therapy with intravenous efgartigimod may be effective for patients with refractory GBS. However, we cannot rule out the possibility that the initial IVIG administered to our patient may have worked or that this was the natural course of his GBS. Further studies are necessary to establish the precise efficacy of efgartigimod for patients with GBS.

The supply of immunoglobulin products is limited due to their provision by volunteer blood donors, and these products are still in short supply throughout Japan, especially since the number of blood donors decreased after 2020 and the Covid-19 pandemic. The supply of IVIG is also limited due to its various indications, including CIDP and Kawasaki disease. In this regard, the supply of efgartigimod remains consistent. Other IVIG drugs take more time to exert their effects, whereas efgartigimod is relatively fast-acting and can help in early rehabilitation. The main problem with plasmapheresis therapy is its invasiveness, and the implementation of this therapy may not be viable in certain contexts. The administration of efgartigimod is expeditious due to its minimally invasive nature and ease of use. As its mechanism is very similar to that of plasmapheresis, efgartigimod acts very quickly, and early amelioration is achieved compared to IVIG and other immunotherapies. It is anticipated that the efficacy of efgartigimod in GBS will be enhanced in the future.

## Data Availability

The raw data supporting the conclusions of this article will be made available by the authors, without undue reservation.
